# ﻿Labeled 2D images from 3D models of museum specimens: the impact of Structure from Motion modeling parameters on rendered 2D images

**DOI:** 10.3897/zookeys.1266.160408

**Published:** 2026-01-15

**Authors:** Blair Mirka, Christopher Lippitt

**Affiliations:** 1 Department of Geography and Environmental Studies, University of New Mexico, Albuquerque, New Mexico, USA University of New Mexico Albuquerque United States of America

**Keywords:** Machine learning, macro-photogrammetry, SfM, synthetic data

## Abstract

Macro-photogrammetry using Structure from Motion (SfM) is widely used in museums and biorepositories to create high-resolution 3D models for educational outreach and proxy specimen access. These models can enable a wide range of analysis, including serving as a latent source of training data for species inventory approaches that leverage machine learning for automated interpretation of 2D imagery. To assess the potential for these models to generate accurate 2D representations, this study investigates how the resulting 3D models’ geometric and texture accuracy is impacted by key photogrammetric parameters, specifically horizontal image overlap, vertical image overlap, and focus stacking, emphasizing the impacts on rendered 2D images. In our study, we find that focus stacking, which is commonly assumed to enhance accuracy, provides limited to no benefits for machine learning purposes when compared to an original image. Ten ground beetle specimens of varying shapes, sizes, and colors, including beetles with iridescent carapaces, were modeled using SfM. From these models, both geometric and texture (external coloration and patterning) accuracy were quantitatively assessed. Geometric accuracy was evaluated using the mean absolute error (MAE), comparing modeled and actual specimen measurements across five morphometric features (elytra length, the elytra width, the lengths of the second and third antenna segments, and the length of the first and second tibia). The accuracy of the 2D render texture was measured using % RGB similarity and structural similarity index measure (SSIM) comparing the original images to rendered images. Focus stacking has traditionally been considered essential for improving overall image sharpness within 3D specimen models, however it represents a major bottleneck in the 3D digitization workflow as it requires multiple images per view angle and requires a considerable amount of time and resources to successfully create a 3D model. Results from this study indicate that not only are these methods unnecessary but they lose image fidelity when renders are compared to their original 2D image. Image capture parameters such as horizontal and vertical overlap were found to have a significant impact on model geometric accuracy and rendered 2D image quality, with the overall best-performing method in terms of geometric and texture accuracy as well as model creation consistency, using non-focus-stacked images captured at five vertical angles, with a 20° rotation between each vertical imaging line and 11.25° horizontally between scans. These results challenge the necessity of focus stacking as a blanket, best practice for all use cases in contemporary 3D modeling workflows, indicating that forgoing focus stacking is a potentially beneficial and resource-efficient method of rendering 3D specimen models intended to produce accurate 2D renderings such as those used as training data for deep-learning algorithms.

## ﻿Introduction

Natural history collections and biorepositories contain massive amounts of biological information through the collection of information-rich specimens. These specimens can provide insight into the past, present, and future of biodiversity. This includes documentation of long-term ecological changes, including morphological shifts and range expansions in response to climate change (MacLean et al. 2018; Melbourne and Goodkin 2024). This is particularly true for entomological collections where there is a large diversity of species, and specimens are easily collected and stored. Large repositories have entomological collections of specimens numbering in the millions with the Smithsonian alone housing over 35 million specimens and more than 128,000 type specimens that serve as invaluable taxonomic references (Smithsonian 2024).

However, their utility is fundamentally limited by their accessibility. Specimen access has historically been limited to in-person contact, whether at the host institution or through the transport of the specimen to the interested party. This model of accessibility imposes a cost to specimen use both in time and money, while also increasing the risk to the specimen through transport and handling. To overcome this limitation, there have been sustained efforts to digitize natural history collections (Blagoderov et al. 2012; Guidi et al. 2015; Hedrick et al. 2020). The digitization of these collections represents a unique opportunity to use these resources as high-quality and readily available training data for deep learning architectures for automated species identification.

A fundamental challenge to the use of deep learning for automated species identification is the training dataset itself, where rare or elusive species may not have sufficient training data for the model to learn from, reducing model performance for these classes. Natural history and biorepository collections represent the single largest source of expert-labeled examples which can be integrated into deep learning models. The development of automated species identification methods would not only help address the backlog of unidentified specimens in museums and biorepositories caused by the ongoing taxonomic crisis (Engel et al. 2021), but it would also enhance the efficiency of large-scale ecological surveys such as those conducted by the National Ecological Observation Network (NEON 2024). By enabling rapid and accurate species identification, these systems hold the potential to facilitate near real-time biodiversity monitoring, improving conservation efforts and ecological research (Høye et al. 2021)

While 2D imaging has been the standard for biological specimen digitization, it has fundamental limitations on what information it can provide, showing only a few view angles, limited information on size and volume, and incomplete structural details. This has led researchers to turn to 3D models for more impactful analysis (Nguyen et al. 2014; Davies et al. 2017; Qian et al. 2019). These 3D models represent the specimen with high fidelity and can be used to synthesize 2D images from any view angle. Expertly labeled images of specimens that provide views from multiple perspectives have proven to be critical for morphological analysis. This is information is readily available when working with 3D models and these digitization efforts have enabled novel analysis (Davies et al. 2017). While the uses of these 3D datasets are growing, their ability to generate high quality synthetic training data for machine learning has yet to be fully explored.

Photogrammetric methods, specifically Structure from Motion (SfM), is a popular choice for 3D specimen modeling due to the low overhead cost and ease of use (Medina et al. 2020). High-quality digital models can be created with standard museum equipment such as a rotating stand, photography lighting, and a high-quality digital camera and lens. The process of macro-photogrammetry may raise setup costs slightly due to the high-magnification lens needed to capture fine details on small subjects (Lastilla et al. 2019; Medina et al. 2020) but still represents an accessible price point for most institutions. Macro-photogrammetry may also require an additional acquisition and processing step, called focus stacking, which requires taking images at multiple depths of fields and then combining them into one image where all parts of the specimen are in focus (Gallo et al. 2014). Lastilla et al. (2019) tested various sensors— ScanRider 1.2 laser scanner, iPad Air 2, and Canon EOS 1200D—with different lenses for creating 3D models of small, complex inscribed objects. Images were captured with overlapping paths in natural or common artificial light, stacked with Helicon Remote (Helicon Soft, 2023), and processed in Agisoft Photoscan. The Canon EOS 1200D with a 100 mm lens produced models with high geometric accuracy (on average 0.1 mm) while also preserving features not captured using the laser scanner.

As technology has advanced and the uses of 3D models have continued to expand, a reevaluation of what is considered best practices for the generation of 3D models for small specimens is warranted, particularly in the context of computer vision applications. Using ground beetles in the family Carabidae, this research aims to understand how different imaging patterns associated with the photogrammetric generation of 3D models affect the geometric and texture (meaning the external color and pattern of a specimen) accuracy of 2D image renders. This is done by looking at the impact of two primary variables in macro photogrammetric scanning (Qian et al. 2019): imaging density/redundancy and focus stacking. A series of controlled imaging patterns using varying densities (i.e., horizontal and vertical overlaps) and with focus stacking or without focus stacking, were conducted and 3D specimen models were generated for each imaging pattern. These models were then rendered and compared to the original 2D images to assess how the modeling process affected the final renderings and their fidelity to the original specimens. The research questions addressed in this study are as follows:

1. What are the best imaging practices for macro photogrammetry for biological specimens in the context of synthetic image generation for computer vision?
2. How do photogrammetric imaging patterns and procedures, specifically image density/redundancy and focus stacking, affect the geometric and texture accuracy of rendered 2D images?
3. How do the rendered images compare quantitatively to the original images, and how do 3D modeling choices influence synthetic 2D model output fidelity?

## ﻿Methods

**Figure 1.**
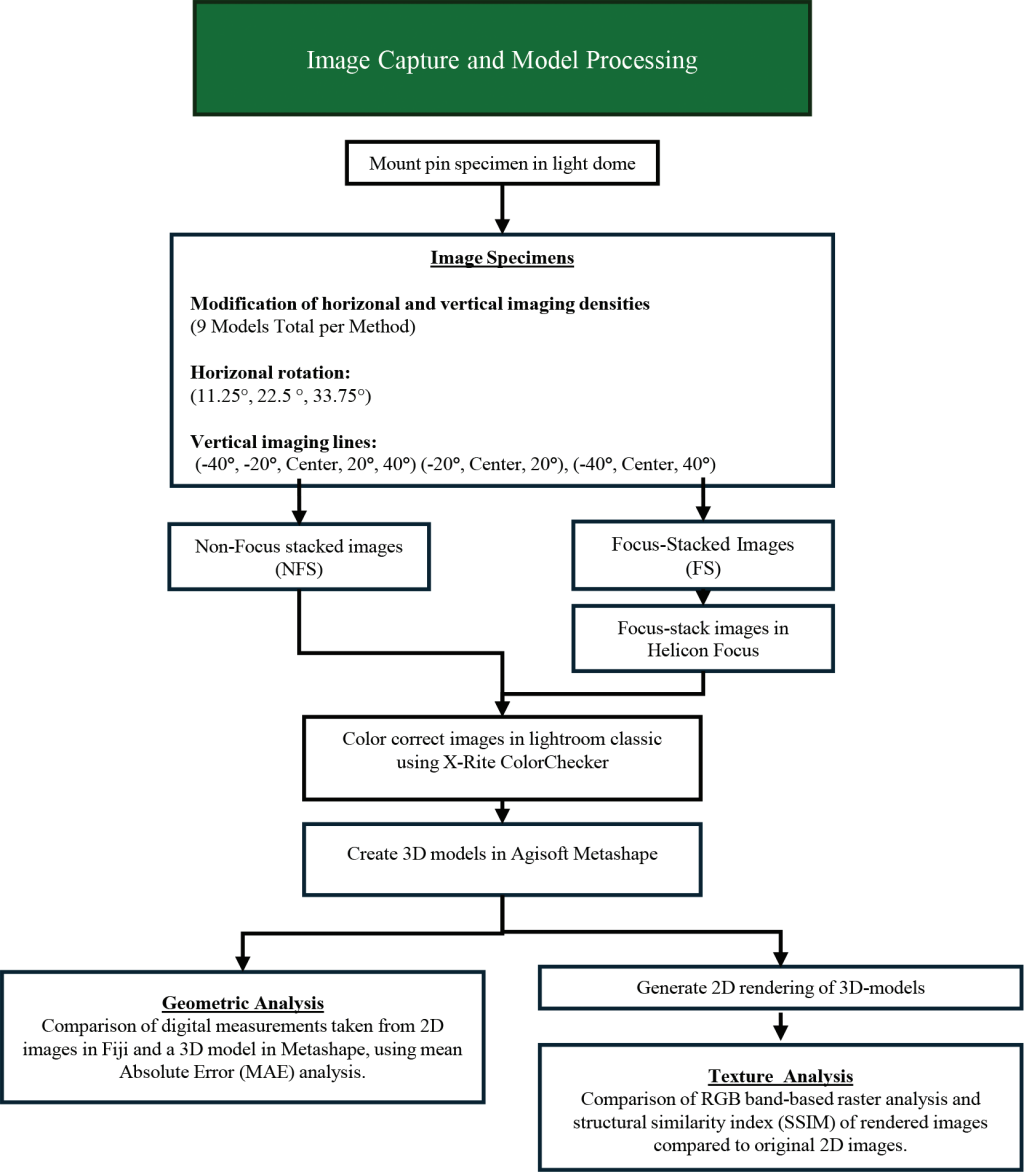
A step-by-step workflow diagram illustrating the processes and methods used in this study, from initial data collection through image collection to the final quantitative analysis of geometric and texture accuracy.

The following section details the procedures used for image capture, image processing, photogrammetric reconstruction, and image rendering. Additionally, this section discusses the metrics used to assess geometric and texture accuracy. Fig. 1 presents a workflow of these steps, which are: imaging setup, image capture using Focus Stacking (FS) and Non-Focus Stacking (NFS) techniques and testing varying imaging densities (i.e. horizontal and vertical specimen rotation between scans).

### ﻿Specimens

**Figure 2.**
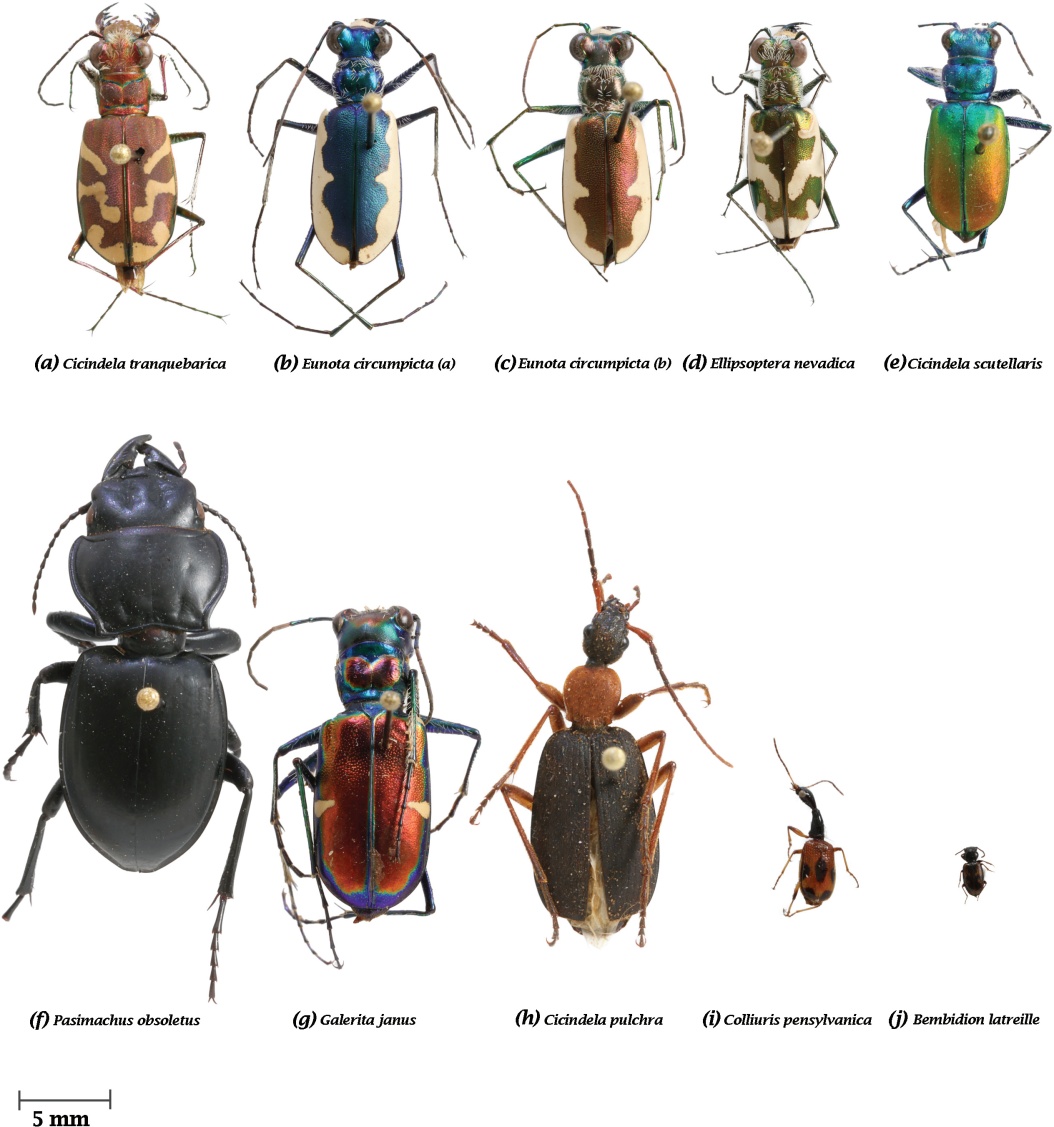
Ten beetles selected from the University of New Mexico’s Museum of Southwestern Biology Division of Arthropods for this study. a. *Cicindela tranquebarica*; b. *Eunota circumpicta (a)*; c. *Eunota circumpicta (b)*; d. *Ellipsoptera nevadica*; e. *Cicindela scutellaris*; f. *Pasimachus obsoletus*; g. *Cicindela pulchra*; h. *Galerita janus*; i. *Colliuris pensylvanica*; j. *Bembidion latreille*.

Ten ground beetle species were selected from the University of New Mexico’s Museum of Southwestern Biology Division of Arthropods to represent a variety of taxonomic variables including size (~4–30 mm), color, and morphology: *Cicindela tranquebarica* (Fig. 2a), *Eunota circumpicta* (Fig. 2b), *Eunota circumpicta* (Fig. 2c), *Ellipsoptera nevadica* (Fig. 2d), *Cicindela scutellaris* (Fig. 2e), *Pasimachus obsoletus* (Fig. 2f), *Galerita janus* (Fig. 2g), *Cicindela pulchra* (Fig. 2h), *Colliuris pensylvanica* (Fig. 2i), *Bembidion latreille* (Fig. 2j)

### ﻿Image capture and pre-processing

Images were captured using a Canon EOS R7 equipped with a 100 mm f/2.8 L macro IS USM lens. The camera has a 32.5-megapixel APS-C CMOS (22.3 mm × 14.8 mm) sensor. All images were taken in JPEG format (24-bit, 8-bit per band) to ensure compatibility across all processing steps. To capture a 360-degree view and to maintain consistent lighting of the specimen, a modified version of the scAnt (Plum and Labonte 2021) illumination dome was used (https://github.com/evo-biomech/scAnt). Components were redesigned to allow the dome to be fully 3D printed, and the stepper motors were replaced with simple gears for manual rotation (Mirka 2024). Two semi-transparent white half-domes with LED strip lights (CRI = 93, 6000 K) provided even, and neutral subject illumination. Specimens were pinned on a semi-arc mount, which allowed for an ~80° rotation along the *y*-axis. A 5 mm 3D printed cube (±0.1 mm) was provided for scale. A full 90° rotation was avoided to minimize interference with the pin head and the scale cube which obscured visibility at higher angles. All components were printed using Bambu Lab P1S 3D printer (Bambu Lab 2023) with Polylactic acid (PLA) filament.

Consistency of color was maintained across images using the X-Rite ColorChecker (X-Rite 2023). A neutral gray patch was used for white balance, and red, green, and blue patches were used as calibration targets. All image adjustments were made in Adobe Lightroom Classic (Adobe Inc. 2024) to the approximate sRGB values.

Pinned specimens were mounted upright and imaged with the camera level with the specimen (0°). Specimens were rotated 11.25° horizontally between images to complete a 360° series, 32 images in total. This process was repeated at angles of ±20° and ±40, producing 160 images (or image stacks). Image capture was semi-automated using Helicon Remote (Helicon Soft 2023). NFS images were taken with a f-stop of f/22 and an ISO of 100 to maximize depth of field. FS images were taken with a f-stop of f/8 and a 100 ISO and stacked (4–11 individual images, depending on the size of the specimen). Image stacks were processed using Helicon Focus Pro v. 8.2.2. (Helicon Soft 2023). The Helicon Focus rendering “method b” was used to best preserve original color and contrast (Lastilla et al. 2019), with Radius (8 px) and Smoothing (3 px) kept at the default settings.

### ﻿SfM processing

**Figure 3.**
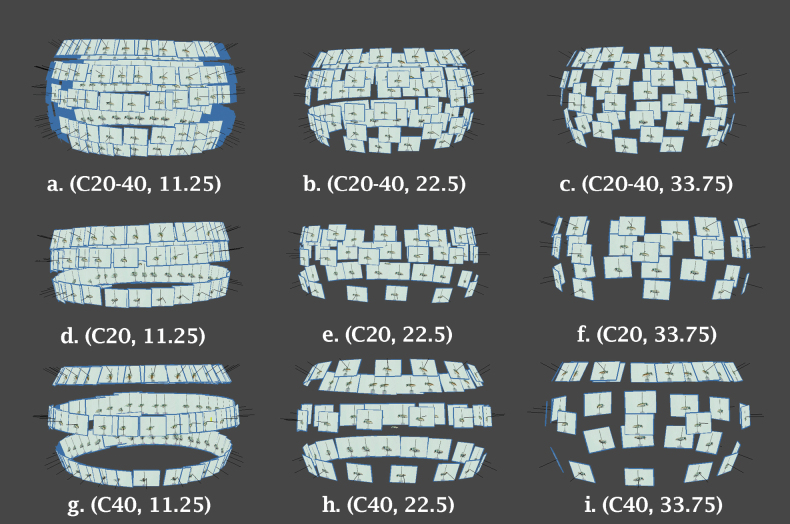
Image density is impacted both by horizontal as well as vertical overlap: image density resulting from: a. 11.25° horizontal rotation and C±20-40 vertical tilt; b. 22.25° horizontal rotation and C±20-40 vertical tilt; c. 33.75° horizontal rotation and C±20–40 vertical tilt, d. 11.25° horizontal rotation and C±20 vertical tilt, e. 22.25° horizontal rotation and C±20 vertical tilt; f. 33.75° horizontal rotation and C±20 vertical tilt, g. 11.25° horizontal rotation and C±40 vertical tilt; h. 22.25° horizontal rotation and C±40 vertical tilt; i. 33.75° horizontal rotation and C±40 vertical tilt.

Once color correction was applied and images stacked as necessary, images were imported to Agisoft Metashape v. 2.1.1.1 (Agisoft Metashape Professional 2024) for photogrammetric processing. To test the effects of imaging parameters, 18 different imaging scenarios were processed varying FS and NFS images, degrees of horizontal rotation between images (11.25°, 22.5°, 33.75°), and vertical tilt (−40°, −20°, Center, 20°, 40°), (−20°, Center, 20°), and (−40°, Center, 40°), hereafter referred to a C±20–40, C±20 and C±40. Table 1 summarizes the variation tested for each variable. Images were removed at specific intervals (every other or every third image) or according to the imaging line parameters (20° or 40°) to simulate variations in imaging densities. Fig. 3 shows how imaging density is impacted by the modification of these variables.

**Table 1.** Three primary variables were tested: imaging style, horizontal rotation, and vertical tilt. Eighteen models were created for each specimen by combining these parameters.

Imaging style	Horizontal rotation	Vertical tilt
Focus stacked (FS)	11.25°, 22.5 °, 33.75°	C±20-40, C±20, C±40
Non-focus stacked (NFS)	11.25°, 22.5 °, 33.75°	C±20-40, C±20, C±40

Once imported, the Metashape background masking function was applied to all images, both reducing processing time and noise by focusing on only areas of interest. During the alignment phase, stationary tie points, or tie points that do not move between images, were also omitted, further reducing any background information not excluded by the mask. The alignment parameters were set to “high”, the key point limit set to 240,000, and the tie point limit set to 0 (i.e. no limit) for all models (Mirka et al. 2025). Scale bars were generated using the 3D-printed 5 mm cube.

Mesh models were built directly from depth maps generated during the initial alignment phase. To create realistic texture, a UV map (‘U’ and ‘V’ refer to axes representing the horizontal and vertical directions in the 2D space of the texture map) was created to unwrap the 3D mesh into 2D space for accurate texture placement. Coloration and surface detail was derived from the original images and quality was further refined using Metashape’s “Mask Defocus Areas” which is used to prioritize in-focus regions for the final texture mosaic improving overall sharpness. Textures were generated using the generic mapping mode, which produced organized, easily editable files. In total, 180 models (18 per specimen for each of the 10 specimens) were created.

The pin and the scale cube were edited from the mesh within Metashape and the resulting holes in the top and bottom of the model were filled using the “close hole” feature, which repairs the mesh by calculating the surrounding vertices, edges, and faces and interpolates the surface area, covering the gap. If the “close hole” feature resulted in ragged edges or irregular geometry, the “smoothing” function, which averages the surface normal and vertex positions of the mesh thereby smoothing the model, was applied to the area surrounding the repaired hole creating more even surface. Within the texture file the location of the pin had no texture value resulting in a black spot on the model, this was manually edited out in Adobe Photoshop (Adobe Photoshop v. 24.1.1) using the clone tool to use the surrounding pixels to estimate what the area looked like before pin placement.

Models that were successfully created, defined here as containing minimal apparent distortion or missing body segments upon visual inspection, were then exported as .obj files and processed to render 2D views of the specimens. Rendered 2D images were generated from specific viewing angles: top (20° or 40°), middle (0°), and bottom (−20° or −40°) of the model. These renderings, produced in Metashape, preserved the original lighting conditions and camera orientations from the source images, enabling direct comparisons to the images used to construct the model. Models were rendered using perspective projection for a realistic sense of scale and depth using depth distortion (foreshortening).

### ﻿Analysis of 3D model and 2D rendering accuracy

**Figure 4.**
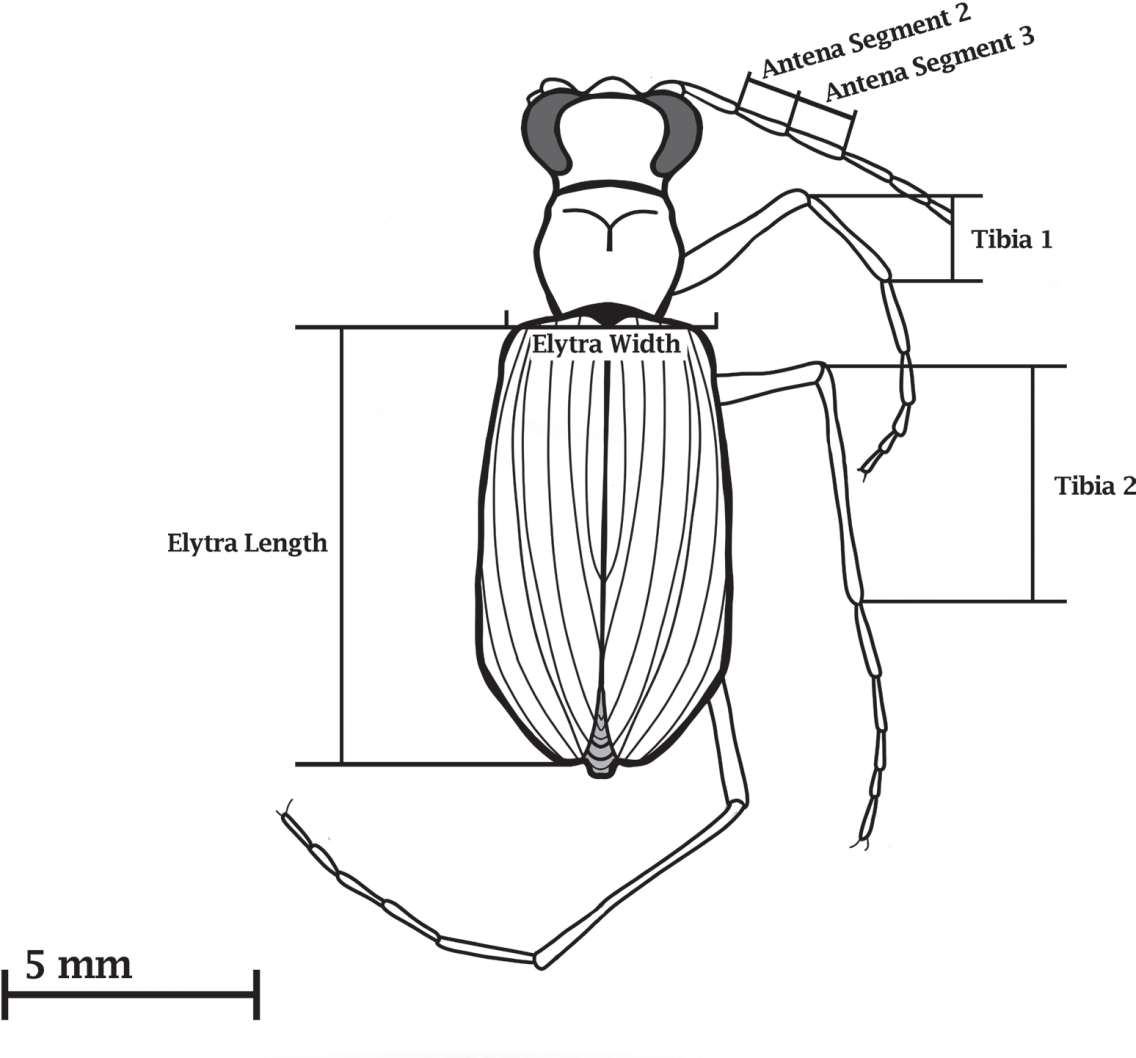
A depiction of where measurements were taken for each beetle. Measurements included the length of the second and third segments of the antennae, the length and width of the elytra, and the length of the first and second tibiae.

Once the 3D models and 2D renderings were complete, analysis was performed to quantify the model’s accuracy in terms of geometry (i.e. morphometric) and texture (i.e. coloration and pattern). To determine the morphometric accuracy, a series of measurements were taken to quantify various body features (Fig. 4). These six features were the elytra length, the elytra width, the lengths of the second and third antenna segments, and the length of the first and second tibia following Elek et al. (2014).

Digital measurements were taken from the original images using Fiji: ImageJ, an open-source biological image analysis software, which has a precision of 0.001 mm (Schindelin et al. 2012). To avoid errors with angular misalignment, images from which measurements were taken were selected based on the criteria that the measured feature was parallel to the camera sensor plane. Measurements of the 3D model were taken within Metashape using the same camera alignment to maintain orientation and preserve the view angle. In cases where a body segment was not properly modeled (i.e., missing or incomplete), the measurement value was omitted. The Mean Absolute Error (MAE) was calculated across all measurements for each specimen to provide a comprehensive metric of geometric error. This allowed for direct comparison across modeling methods and specimens and offered an overall indication of model quality regardless of error directionality.

To assess texture accuracy, rendered images were compared to the corresponding original images from the same perspective. The “Select and Mask” feature within Photoshop was used to clip both the original and rendered image to the same spatial extent, isolating the specimen for comparison and reducing any background bias. Three distinct images, one from the center (0°), top (20° or 40°), and bottom (−20° or −40°) were used to ensure key features (elytra, setae, etc.) were included in rendered images. The average error associated with these three views was used to assess overall texture accuracy for each specimen.

Two different metrics were used to assess the similarity of the original image to the image rendered from the 3D model. The first metric used was an RGB band-based raster analysis calculating the percent similarity between the rendered image (*A*) and the original image (*B*) on a pixel-by-pixel basis, then averaging the errors for the channel (*C*, where *C* is {R, G, B}) the absolute difference at each pixel location (*i, j*) is defined as:

Δ_C_ (x,y) = |A_C_ (i,j) − B_C_ (i,j)|

Overall similarity for each band (i.e. RGB) was calculated as a percentage for each channel *C* based on the ratio of total observed differences to the maximum possible difference. The similarity between the two images is defined as:

$${\text{ Similarity }}_{C}=\left(1-\frac{\Sigma_{i,j}\Delta_{\left(i,j\right)}}{255\times \text{ total pixels }}\right)\times 100$$

Where 255 × total pixels is the maximum difference possible if all pixels within the image were maximally different. The overall RGB similarity is obtained by averaging the similarity percentages across all three channels:

$${\text{ Similarity }}_{RGB}=\frac{1}{3}\sum_{i\in \{R,G,B\}}{\text{ Similarity }}_{C}$$

To assess the overall model rendering performance, overall image similarities in the three view perspectives were averaged to achieve a final model accuracy assessment:

$$\text{ Overall Image Similarity }=\frac{1}{3}\sum_{i\in \{\text{ Top,Center,Bottom }\}}{\text{ Similarity }}_{RGB}$$

**Figure 5.**
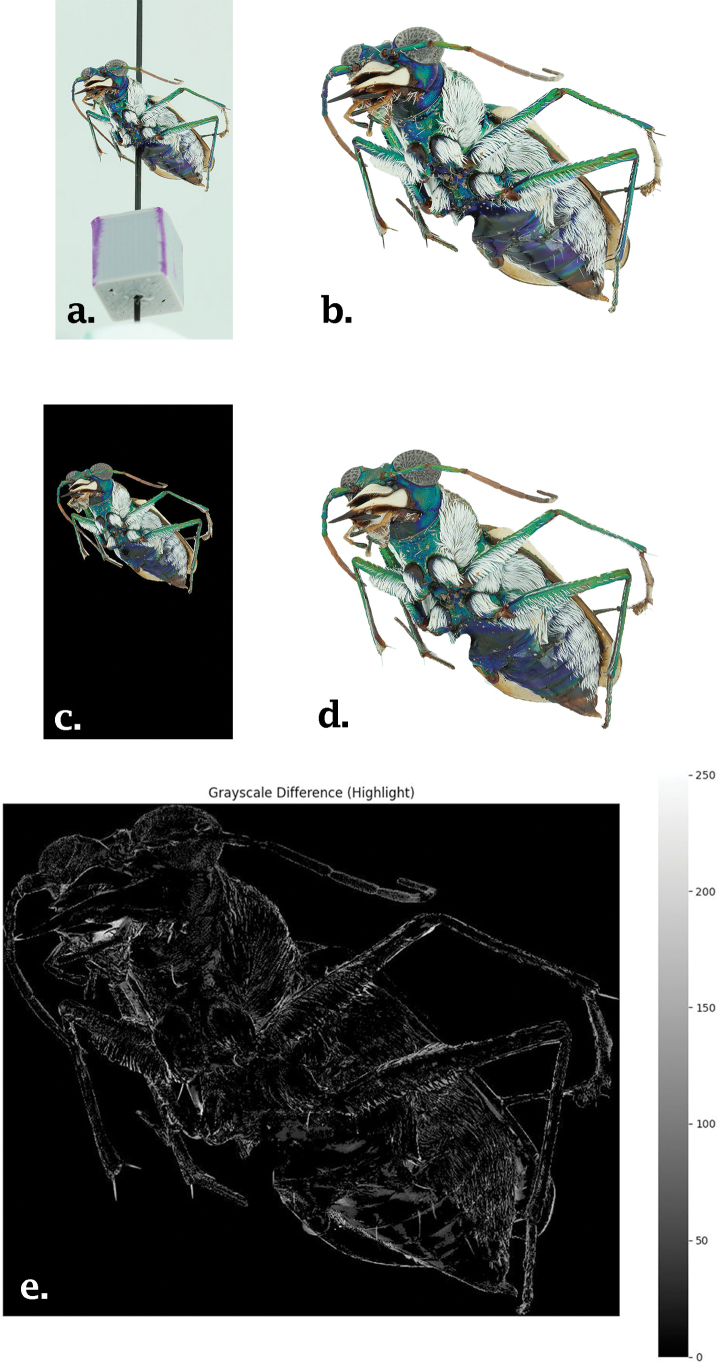
The process compares the (c) rendered model image with the (a) original image by masking both to an identical extent (b, d). This allows for an assessment of differences across each of the (e) RGB color bands.

Difference maps were generated using the differenced values between pixels to visually highlight areas of significant error, specifically where difference values exceeded a threshold of 30. This threshold was chosen to reduce noise and emphasize only regions with significant errors. The highest pixel values (i.e. largest difference) indicate the locations of the largest differences (Fig. 5).

The second metric used to assess texture accuracy was the structural similarity index (SSIM) (Wang et al. 2004). This is a perceptual metric that quantifies the similarity between two images by considering not only coloration but also luminance (brightness), contrast, and structure. This aligns more closely with human perception of images (Wang et al. 2004). The SSIM index between two images, *x* and *y*, is defined mathematically as:

$$\mathrm{SSIM}(x,y)=\frac{\left(2_{\mu_{x}\mu_{y}}+C_{1}\right)\left(2\sigma_{xy}+C_{2}\right)}{\left(\mu_{x}^{2}+\mu_{y}^{2}+C_{1}\right)\left(\sigma_{x}^{2}+\sigma_{y}^{2}+C_{2}\right)}$$

Where, *µ_x_* and *µ_y_* are the mean pixel values of images *x* and *y*, $\sigma_{x}^{2}$ and

$$\sigma_{y}^{2}$$

are the variance of pixel values in the windows of images *x* and *y*, and $\sigma_{xy}$ is the covariance between *x* and *y.*SSIM values are between −1 and 1, where 1 indicates perfect similarity.

## ﻿Results

**Figure 6.**
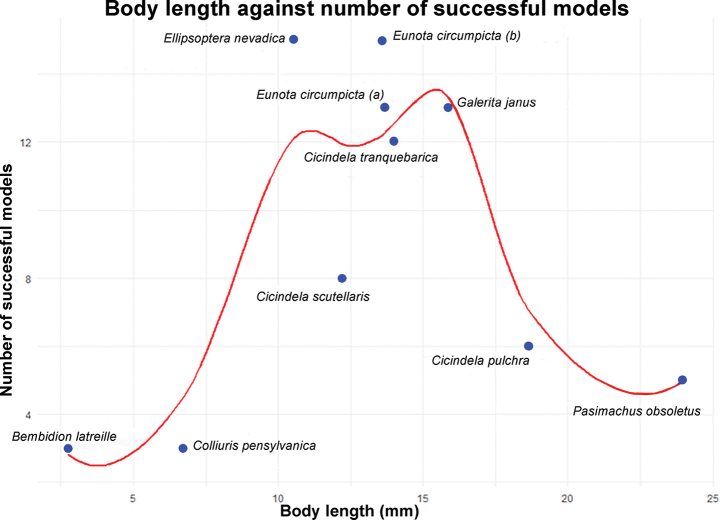
Specimen size was a key factor in determining if a model would be successfully created among model parameters. Outliers on either end of the spectrum resulted in fewer models being successfully created using lower imaging density.

Out of 180 3D models attempted, 93 were successfully created. The number of models successfully create varied across species and modeling parameters. The number of successful models generated was generally related to the size of the specimen, with specimens at either extreme (smaller or larger) showing reduced numbers of successfully created models (Fig. 6). For each specimen, the best model was determined by looking at both geometric and texture accuracy and NFS datasets consistently showed the same or better results as the focus-stacked images (Table 2).

**Table 2.** Imaging parameters (focus stacking (FS) vs non-focus stacking (NFS), horizontal rotation, and vertical tilt) are provided for the highest overall accuracy model.

Specimen	Number of successful models	Body length (excluding antennae and legs) (mm)	Highest overall accuracy model(s) parameters
*Cicindela scutellaris*	8/18	12.20	NFS (C20, 11.25)
*Galerita janus*	13/18	15.85	NFS (C20-40, 11.25)
*Cicindela pulchra*	6/18	18.65	NFS (40, 11.25)
*Pasimachus obsoletus*	5/18	23.96	FS (C20, 11.25)
*Ellipsoptera nevadica*	15/18	10.54	NFS (C20-40, 22.25)
*Bembidion latreille*	3/18	2.75	NFS (C20-40, 11.25)
*Eunota circumpicta* (a)	13/18	13.69	NFS (C20-40, 11.25)
*Eunota circumpicta* (b)	15/18	13.58	NFS (C40, 11.25)
*Cicindela tranquebarica*	12/18	13.98	NFS (C20-40, 11.25)
*Colliuris pensylvanica*	3/18	6.70	NFS (C20-40, 11.25)

In total NFS (C20-40, 11.25) had a 100% success rate with 10 out of 10 models successfully completed. NFS (C20, 11.25) and FS (C20-40, 11.25) had 9 successful models, and all other parameter combinations had 9 or fewer. Table 3 shows the number of models created and the average geometric and texture accuracy per model. Among successfully created models, quality remains relatively consistent across all metrics. Therefore, a key feature of different modeling parameters is their ability to consistently produce successful models across specimens of varying sizes.

**Table 3.** A comparison of all different model parameter combinations reveals the created models exhibit similar quality, with the main differences being observed in model creation success rate. The evaluation metrics used were number of successful models, calculated percentage RGB similarity, SSIM score, and the MAE across measurements in millimeters (mm).

Model parameters	# of successful models	RGB similarity %	SSIM	MAE (mm)
NFS (C20, 11.25)	9	96.57	0.88	0.18
NFS (C20, 22.5)	5	96.34	0.86	0.27
NFS (C20, 33.75)	2	96.24	0.86	0.21
NFS (C40, 11.25)	8	96.60	0.88	0.21
NFS (C40, 22.5)	7	96.39	0.87	0.25
NFS (C40, 33.75)	2	96.22	0.85	0.20
NFS (C20–40, 11.25)	10	96.95	0.89	0.16
NFS (C20–40, 22.5)	8	96.76	0.88	0.20
NFS (C20–40, 33.75)	6	96.36	0.87	0.19
FS (C20, 11.25)	6	96.63	0.84	0.21
FS (C20, 22.5)	1	96.28	0.83	0.12
FS (C20, 33.75)	3	95.63	0.81	0.24
FS (C40, 11.25)	6	96.18	0.83	0.19
FS (C40, 22.5)	3	96.10	0.83	0.20
FS (C40, 33.75)	0	—	—	—
FS (C20–40, 11.25)	9	96.54	0.84	0.21
FS (C20–40, 22.5)	5	96.26	0.83	0.25
FS (C20–40, 33.75)	3	95.97	0.82	0.17

Increased image density both horizontally (11.25°) and vertically (−40°, −20°, 0°, 20°, and 40°) created quality models consistently indicating that high overlap is critical to ensure all models are created successfully. Table 4 shows measurement error by feature. From this it is clear that while certain imaging parameters may be able to successfully create a model it is not guaranteed that key features will be included. Overall, measurement error did not vary dramatically across model parameters. However, some of the lower success rate model parameters show a slightly reduced error when compared to models with higher success rates, which can be attributed to them failing to create models of the more complex specimens, thereby reducing their error by omitting them entirely. More formal statistical testing was not performed due to the uneven number of successful models across imaging conditions which would limit interpretive and statistical value. However, the absolute difference across imaging parameters provides an overview of the trends within the dataset and shows that there is little difference in error across successfully created models.

**Table 4.** MAE for each measured feature and the number of successful measurements across model parameters (successful/total), categorized by imaging method, view angle, and image density.

Model Parameters	Elytra length MAE (mm)	Elytra width MAE (mm)	Antenna segment 2 MAE (mm)	Antenna segment 3 MAE (mm)	First tibia MAE (mm)	Second tibia MAE (mm)
NFS (C20, 11.25)	0.33 (9/9)	0.22 (9/9)	0.05(8/9)	0.04 (9/9)	0.22(8/9)	0.26 (9/9)
NFS (C20, 22.5)	0.37 (5/5)	0.31 (5/5)	0.11 (3/5)	0.08 (4/5)	0.24 (5/5)	0.27 (5/5)
NFS (C20, 33.75)	0.30 (2/2)	0.09 (2/2)	0.09 (1/2)	0.07 (2/2)	0.24 (2/2)	0.45 (2/2)
NFS (C40, 11.25)	0.42 (8/8)	0.30 (8/8)	0.03(8/8)	0.05 (8/8)	0.20 (8/8)	0.28 (8/8)
NFS (C40, 22.5)	0.37 (7/7)	0.26 (7/7)	0.22(7/7)	0.06 (6/7)	0.24 (7/7)	0.20 (6/7)
NFS (C40, 33.75)	0.56 (2/2)	0.12 (2/2)	0.16 (2/2)	0.02 (2/2)	0.17 (2/2)	0.17 (2/2)
NFS (C20-40, 11.25)	0.34 (10/10)	0.15 (10/10)	0.08 (10/10)	0.05 (10/10)	0.15 (10/10)	0.20 (10/10)
NFS (C20-40, 22.5)	0.33 (8/8)	0.24 (8/8)	0.10 (8/8)	0.05 (8/8)	0.23 (8/8)	0.25 (8/8)
NFS (C20-40, 33.75)	0.33 (6/6)	0.28 (6/6)	0.04 (6/6)	0.05 (6/6)	0.21 (6/6)	0.22 (6/6)
FS (C20, 11.25)	0.43 (6/6)	0.32 (6/6)	0.04 (5/6)	0.02 (6/6)	0.21 (6/6)	0.16 (6/6)
FS (C20, 22.5)	0.60 (1/1)	0.22 (1/1)	N/A (0/1)	N/A (0/1)	N/A (0/1)	N/A (0/1)
FS (C20, 33.75)	0.43 (3/3)	0.13 (3/3)	N/A (0/3)	0.11 (1/3)	0.22 (3/3)	0.21 (3/3)
FS (C40, 11.25)	0.37 (6/6)	0.27 (6/6)	0.07 (6/6)	0.02 (6/6)	0.19 (6/6)	0.21 (6/6)
FS (C40, 22.5)	0.18 (3/3)	0.32 (3/3)	0.06 (1/3)	0.04 (2/3)	0.03 (2/3)	0.24 (2/3)
FS (C40, 33.75)	0					
FS (C20-40, 11.25)	0.42 (9/9)	0.23 (9/9)	0.08 (8/9)	0.04 (8/9)	0.25 (8/9)	0.26 (9/9)
FS (C20-40, 22.5)	0.50 (5/5)	0.34 (5/5)	0.04 (5/5)	0.05 (5/5)	0.26 (5/5)	0.30 (5/5)
FS (C20-40, 33.75)	0.23 (3/3)	0.14 (3/3)	0.08 (2/3)	0.06 (2/3)	0.14 (3/3)	0.27 (3/3)

More formal statistical testing was not performed due to the uneven number of successful models across imaging conditions which would limit interpretive and statistical value. However, the absolute difference across imaging parameters provides an overview of the trends within the dataset and shows that there is little difference in error across successfully created models.

## ﻿Discussion

The need for fast and accurate methods of generating 3D models of museum specimens is clear, particularly for machine learning applications. Efficient 3D modeling not only has the potential to facilitate automated species identification but can also increase specimen accessibility. The creation of 3D models allows for more in-depth analysis and interaction compared to traditional 2D imagery. These methods also open the door for novel research techniques (e.g. Nguyen et al. 2014; Davies et al. 2017; Qian et al. 2019), including the creation of synthetic training data for deep-learning classifiers and the opportunity to correct for class imbalances common in ecological datasets. Time and cost-effective generation of expert labeled training data from 3D models has broad implications for large scale ecological monitoring such as NEON and for community-based biodiversity monitoring such as iNaturalist. The importance of these methods is widely seen in the entomological community (Høye et al. 2021) and through other large-scale digitization initiatives such as Big-Bee (https://github.com/Big-Bee-Network). These results indicate that while focus stacking does provide benefits in terms of preservation of fine detail and creation of visually stunning models, in the context of rendering synthetic 2D representations of specimens, there is limited benefit, and the investments in time and resources present a major bottleneck when working towards large-scale 3D digitization initiatives.

To provide an initial assessment on how these findings might manifest in a machine learning environment, a YOLOv8s model was tested using a limited dataset. Synthetic images derived from the 3D models were rendered in Blender v. 4.2 LTS (Blender Development Team 2025) using the most accurate models generated from both the FS and NFS reconstructions of each specimen. To ensure comparability, all synthetic images were rendered from the same perspective and with the same background.

Fifty images were downloaded from GBIF (GBIF.org 2025) for each *Cicindela Scutellaris*, *Cicindela Pluchra*, *Cicindela tranquebarica*, *Ellipsoptera nevadica*, *Enota Circumpicta*, and *Colliuris pensylvanica*. *Pasimachus obsoletus*, *Galerita janus*, and *Bembidion latreille* were excluded from this analysis due to limited publicly available images. The GBIF dataset was split into training (35 images per species), validation (10), and testing (5) subsets.

To test the impact of synthetic data, 10 randomly selected images were removed from the training subset of each class and replaced with synthetic images generated from either the FS or NFS models. *Colliuris pensylvanica* was excluded from this comparison as a complete FS model was not successfully created preventing direct evaluation between the two methods.

Performance metrics for the comparison of YOLO models trained with supplemental datasets are summarized in Table 5. Overall, the models performed similarly when compared to one another, showing preliminarily that both FS and NFS show the potential to be a viable solution when creating supplemental synthetic data.

**Table 5.** Preliminary results on a limited dataset indicate that the difference in NFS and FS is class-dependent, and it is not evident that one method is consistently greatly superior across all classes.

NFS						
Class	Images	Instances	*P*	*R*	mAP50	mAP50-95
All	60	64	0.851	0.757	0.801	0.587
*Cicindela pulchra*	10	10	0.834	0.9	0.918	0.596
*Cicindela scutellaris*	10	10	1	0.796	0.862	0.647
*Cicindela tranquebarica*	10	10	1	0.447	0.668	0.468
*Ellipsoptera nevadica*	10	13	0.571	0.769	0.662	0.461
*Eunota circumpicta*	10	11	0.752	0.727	0.793	0.592
FS						
Class	Images	Instances	*P*	*R*	mAP50	mAP50-95
All	60	64	0.828	0.742	0.843	0.62
*Cicindela pulchra*	10	10	0.789	0.9	0.962	0.655
*Cicindela scutellaris*	10	10	1	0.798	0.825	0.721
*Cicindela tranquebarica*	10	10	0.842	0.536	0.852	0.617
*Ellipsoptera nevadica*	10	13	0.869	0.511	0.687	0.386
*Eunota circumpicta*	10	11	0.694	0.909	0.86	0.599

For the NFS models, precision across all classes within the dataset was only slightly higher than FS models with p = 0.851 and p = 0.828 respectively. Recall across all classes was marginally lower (R = 0.757 and 0.742). The mAP50 across all classes was higher in FS (0.843) compared to NFS (0.801). Class level performance varied indicating that there may be differences in the effectiveness of FS vs NFS at a species level. For example, *Cicindela tranquebarica* showed the most sensitivity to the modeling methods with a mAP50 of 0.825 for FS and a significant decrease when using NFS images with a mAP50 of 0.668. However, most classes showed only minor decreases in accuracy ±0.02–0.07.

Overall, these results suggest that the difference between FS and NFS-trained models were generally minor. Although more rigorous testing will need to be conducted to validate these claims. Trends across classes indicate the model performance is more strongly influenced by species-level detectability trends, meaning variations in body size, coloration or distinguishing features, rather than by FS vs NSF rendered training images. This supports the use of NFS datasets as an efficient alternative to FS when time and resources are limited. While isolated classes show larger shifts (*Cicindela tranquebarica*, mAP50 = - 0.149) this does not imply that there is a consistent disadvantage to using NFS. It is important to note that these results are based on a very limited dataset and should be considered preliminary but promising results. Future research should explore how these patterns change with larger datasets including greater specimen and species diversity.

These results are particularly relevant in the current context of macro photogrammetry where FS is a standard step in the processing of images for use in specimen 3D modeling (Gallo et al. 2014; Brecko and Mathys 2020; Plum and Labonte 2021). FS has many advantages, particularly in areas where fine detail preservation and aesthetics are necessary and need to be prioritized. This includes areas such as morphological research and for public facing 3D models used for community outreach. However, FS fundamentally limits the rate at which 3D models can be generated. For this study, image capture, stacking, and editing from FS image models typically took ~4 h, compared to NFS, which can be completed in 15–20 min. Other research (Brecko and Mathys 2020) has shown similar time investments needed to complete the FS imaging process, even using a fully automated process. There is also the issue of dataset size; the process of FS images requires considerably more individual images to be captured and stored. In this study, FS image datasets can be over 1000 images, whereas NFS image datasets were below 200.

**Figure 7.**
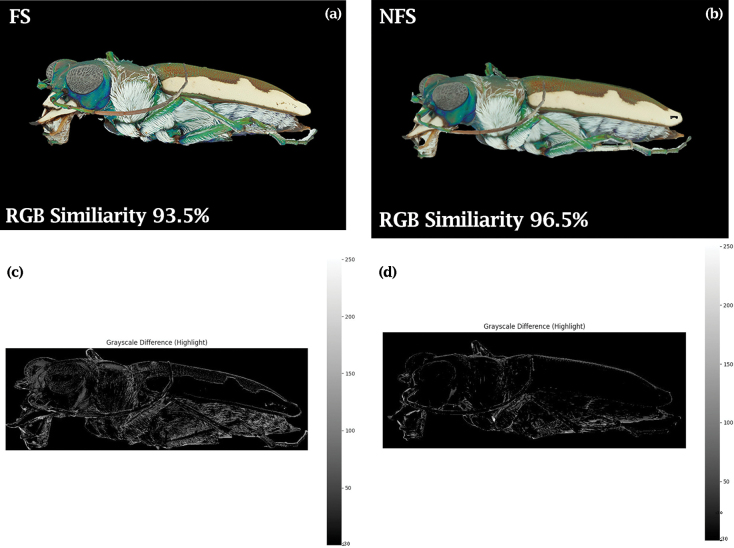
The comparison between the (a) FS render images and the (b) NFS render images shows a clear difference in the aesthetic qualities of the models. However, the NFS image is more comparable to the corresponding source image, indicating some level of texture distortion introduced to the photogrammetric process when using FS images. These differences visualized by the gray scale highlight images for the (c) FS image and the (d) NFS.

The successful use of NFS images in creating high-quality 3D models can increase the practicality of implementing mass 3D digitization efforts by reducing time and storage costs, while improving 2D render realism in the generation of synthetic deep-learning training datasets. This is not to say that FS does not have a role. FS, while not contributing significantly to 2D render accuracy, does create highly detailed texture in the 3D model. For example, the fine details of a beetle’s compound eye can only be captured using FS imaging. However, from a purely pixel difference perspective, the results show minimal variation in texture. This observation has important implications for computer vision applications as it suggests there is little quantitative pixel difference between the render and the original images (Fig. 7).

While FS images consistently provide higher detail in texture, the results of this study indicate they exhibit slightly lower texture accuracy in 2D renderings, suggesting that error is introduced during the rendering process when using focus-stacked images. This may be attributed to error when combining the different depth images or because the depth information is reduced because of consistent focus rather than natural defocusing, creating an artificial lack of depth.

Extreme specimen size proved to limit the model creation success rate and reduce the overall quality of models. A potential reason for this is the need for a higher magnification lens for smaller specimens or, for larger specimens, a wider depth of field may be required. With the same f-stop (in this study f/8), proportionally less of the specimen is in focus for a large specimen compared to smaller specimens. That being said, this reduction in accuracy was not to the extent that the modeling process would be ineffective as a research tool, particularly when using NFS (C20-40, 11.25) model parameters. Fine metric features, such as the lengths of the second and third antenna segments and second and third tibia, were consistently visible and rendered appropriately using NFS (C20-40, 11.25). This modeling parameter shows the highest potential for use in quantitative analysis as it produces reliable, high-quality results.

While this study looked specifically at the use of Metashape for the creation of 3D models for 2D image renders, it is important to note that these methods may not be generalizable across different use cases or taxa. For example, taxonomic identification that relies on or benefits from the identification and delineation of fine features, such as bee wings (Spiesman et al. 2024), may see a decreased accuracy when key taxonomic features are not preserved in NFS images and the resulting models. Additionally, the findings and methods outlined in this paper should be transferable but need to be tested in other imaging scenarios. For instance, the imaging pattern used by Big-Bee (Seltmann et al. 2021), which involves two tilt-imaging patterns, one horizontal, and one vertical, differs dramatically from the concentric orbital pattern used in this study. A review will be necessary to determine if FS remains the optimal method for machine learning given this difference in imaging protocol. Additionally, exploring how methods from these studies can be applied to open-source software like Meshroom would significantly decrease the overhead cost and increase accessibility to smaller institutions. Future work should investigate testing different software and imaging patterns to see if these principles hold true as well as testing different macro specimens which may present their own unique challenges and require their own optimal imaging settings.

Modifying both the lens magnification as well as the aperture settings for smaller and larger specimens respectively should also be explored to understand how these factors impact the geometric and texture accuracy of a range of specimen sizes. Now that the ability to synthesize geometrically and texturally accurate 2D rendering from 3D modeled museum specimens has been demonstrated, the need to evaluate the potential impact of leveraging 3D specimen models to supplement training data for deep-learning models for species identification is clear.

## ﻿Conclusion

This research evaluated synthetic 2D images rendered from 3D models created using macro-photogrammetry to assess their potential as a source of synthetic training data for deep-learning classifiers, using carabid beetles as a case study. This study assessed which imaging parameters resulted in the least amount of geometric and texture error in 2D images rendered from 3D models. Three quantitative methods were used to determine the overall accuracy of images rendered from these models. Geometric accuracy was assessed by comparing measurements from the 3D modeled specimens and those from the actual specimen. Texture accuracy was assessed by comparing pixel values between natural and synthesized 2D images. Using these assessments, the overall best-performing method used non-focus-stacked images, captured at five angles, with a 20° vertical rotation between each imaging line and the specimen rotated 11.25° horizontally between acquisitions. These results show that labeled 2D images can be rendered easily and efficiently from 3D models of museum specimens. A preliminary test of a YOLOv8s model found that there is not a major difference in the use of using rendered 2D image from FS images compared to NFS images. This has profound implications for the potential of 3D specimen models to serve as a massive and accessible source of expert-labeled synthetic training data for computer vision seeking to expand biodiversity models to include rare or elusive morphologies.

## ﻿Additional information

### Use of AI

No use of AI was reported.

### Author ORCIDs

Blair Mirka https://orcid.org/0000-0003-4564-1242

Christopher Lippitt https://orcid.org/0000-0001-7979-7857

### Data availability

All of the data that support the findings of this study are available in the main text.
